# Analysis, Optimization and Verification of Illumina-Generated 16S rRNA Gene Amplicon Surveys

**DOI:** 10.1371/journal.pone.0094249

**Published:** 2014-04-10

**Authors:** Michael C. Nelson, Hilary G. Morrison, Jacquelynn Benjamino, Sharon L. Grim, Joerg Graf

**Affiliations:** 1 Department of Molecular and Cell Biology, University of Connecticut, Storrs, Connecticut, United States of America; 2 Josephine Bay Paul Center, Marine Biological Laboratory, Woods Hole, Massachusetts, United States of America; Charité, Campus Benjamin Franklin, Germany

## Abstract

The exploration of microbial communities by sequencing 16S rRNA genes has expanded with low-cost, high-throughput sequencing instruments. Illumina-based 16S rRNA gene sequencing has recently gained popularity over 454 pyrosequencing due to its lower costs, higher accuracy and greater throughput. Although recent reports suggest that Illumina and 454 pyrosequencing provide similar beta diversity measures, it remains to be demonstrated that pre-existing 454 pyrosequencing workflows can transfer directly from 454 to Illumina MiSeq sequencing by simply changing the sequencing adapters of the primers. In this study, we modified 454 pyrosequencing primers targeting the V4-V5 hyper-variable regions of the 16S rRNA gene to be compatible with Illumina sequencers. Microbial communities from cows, humans, leeches, mice, sewage, and termites and a mock community were analyzed by 454 and MiSeq sequencing of the V4-V5 region and MiSeq sequencing of the V4 region. Our analysis revealed that reference-based OTU clustering alone introduced biases compared to *de novo* clustering, preventing certain taxa from being observed in some samples. Based on this we devised and recommend an analysis pipeline that includes read merging, contaminant filtering, and reference-based clustering followed by *de novo* OTU clustering, which produces diversity measures consistent with *de novo* OTU clustering analysis. Low levels of dataset contamination with Illumina sequencing were discovered that could affect analyses that require highly sensitive approaches. While moving to Illumina-based sequencing platforms promises to provide deeper insights into the breadth and function of microbial diversity, our results show that care must be taken to ensure that sequencing and processing artifacts do not obscure true microbial diversity.

## Introduction

The field of microbial ecology relies on knowledge about the structure and composition of microbial communities as a foundation for understanding their role and function. Culture-independent analyses, which allow the identification of species that are recalcitrant to cultivation, continue to have a large impact on our understanding of microbial communities since the first studies of 5S rRNA sequences by Stahl et al. in the mid 1980s [1], [2]. While many consider full length sequences generated by Sanger sequencing of 16S rRNA clone libraries to be the gold standard for phylogenetic analysis, even the largest studies typically analyzed no more than a few hundred to a thousand sequences for each sample due to the costly and labor intensive process this method entails [3]–[5]. In the early 2000s, the development and commercial availability of high-throughput sequencing platforms capable of producing hundreds of thousands to millions of sequences per run at a significantly lower cost than Sanger sequencing led to a revolution in the field of microbial ecology.

Microbial ecologists quickly adopted high-throughput pyrosequencing instruments produced by Roche 454 Life Sciences for sequencing 16S rRNA genes, which led to the discovery of what has been termed the “rare biosphere” and provided a deeper and more thorough view of the composition of a vast number of microbial communities from a wide range of habitats [6]–[10]. Since its introduction, most investigators have preferred 454 pyrosequencing for microbial diversity projects due to the longer read lengths that the 454 pyrosequencing platform provided relative to competing sequencing instruments from Illumina and others. While capable of producing longer reads lengths than competing technologies, 454 pyrosequencing produces datasets that exhibit characteristic errors associated with insertions/deletions (indels) in stretches of identical nucleotides (homopolymers) [11]. These systematic errors must be removed or corrected using time consuming and computationally intensive software packages prior to further analysis [12]–[14].

Compared to 454 pyrosequencing, the Illumina sequencing-by-synthesis (SBS) methodology has a lower per-base error rate and is not as susceptible to indel errors in homopolymer stretches [15], [16]. The significantly higher sequence quality of Illumina generated sequences, combined with a much lower cost per sequence compared to 454 pyrosequencing, has spurred a number of researchers to develop strategies to sequence 16S rRNA gene amplicons using Illumina systems [17]–[22]. Although initial studies suggested that Illumina-based 16S sequencing produced data of lower quality than 454 pyrosequencing [19], adjustments to the library preparation and sequencing protocols have produced datasets with significantly higher quality than 454 pyrosequencing [18], [22]. While Illumina instruments historically generated short sequences of 30–100 bp, increases in the maximum read length on the Illumina MiSeq platform [2×300 bp paired end sequencing as of this writing) allow the sequencing of amplicons of similar length to those traditionally used in 454 pyrosequencing studies. Additionally, the length and quality of Illumina sequenced amplicons can be increased by aligning and combining each set of paired end reads into a single contig, a process generally referred to as read merging. This allows researchers using the Illumina MiSeq to produce merged sequences with an average length similar to those generated by 454 pyrosequencing but of significantly higher quality and at a lower cost per sequence [17], [22], [23].

While some previous studies have compared the results of 454 pyrosequencing and Illumina sequencing for both metagenomic libraries and 16S amplicons [24]–[26], these studies mostly focused on comparing beta diversity measures to see if the two sequencing technologies produced similar comparisons between different samples. As such, finer details concerning whether Illumina-based 16S sequencing can serve as a replacement for users currently using 454 pyrosequencing have yet to be fully explored. In this study we generated amplicon libraries of the V4-V5 hyper-variable regions of the 16S rRNA gene for 6 natural microbial communities and a synthetic mock community using the same 16S rRNA gene template primers, which were sequenced using either a 454 GS FLX or Illumina MiSeq. Additionally, libraries for the V4 hyper-variable region alone were generated and sequenced on the MiSeq using the protocol described by Caporaso et al. [18] We examined multiple combinations of data processing methods involving OTU clustering and chimera detection to identify a combination that provides both processing efficiency and accuracy. Using this processing method we analyzed the resulting datasets and compared the results of alpha and beta diversity analyses to evaluate whether the choice of sequencing platform led to significant differences that could bias the interpretation of the results.

## Materials and Methods

### Sample descriptions

We chose five samples, representing diverse host-associated microbial communities, for analysis: stool from an adult human (sample Human), contents of the intestinum of the medicinal leech *Hirudo verbana* (sample Leech), contents from the small intestine of a healthy mouse (sample Mouse), the non-adherent microbial fraction obtained from rumen contents of a dairy cow (sample Rumen) and the hindgut contents from the eastern subterranean termite *Reticulitermes flavipes* (sample Termite). Mixed liquor from the municipal waste water treatment facility located on the University of Connecticut, Storrs campus (sample Sewage) was included as a complex, high-diversity environmental microbial community. We also included a synthetic mock community (sample Mock) which was developed by the Human Microbiome Project (HMP) and includes the following 20 bacterial species in equal concentration according to ribosomal copy number: *Acinetobacter baumannii* str. 5377, *Actinomyces odontolyticus* str. 1A.21, *Bacillus cereus* str. NRS 248, *Bacteroides vulgatus* str. NCTC 11154, *Clostridium beijerinckii* str. NCIMB 8052, *Deinococcus radiodurans* str. R1 (smooth), *Enterococcus faecalis* str. OG1RF, *Escherichia coli* str. K12 substr. MG1655, *Helicobacter pylori* str. 26695, *Lactobacillus gasseri* str. 63 AM, *Listeria monocytogenes* str. EGDe, *Neisseria meningitidis* str. MC58, *Propionibacterium acnes* str. KPA171202, *Pseudomonas aeruginosa* str. PAO1-LAC, *Rhodobacter sphaeroides* str. ATH 2.4.1, *Staphylococcus aureus* TCH1516, *Staphylococcus epidermidis* FDA str. PCI 1200, *Streptococcus agalactiae* str. 2603 V/R, *Streptococcus mutans* str. UA159, and *Streptococcus pneumoniae* str. TIGR4. For the 454 library, an earlier version of the HMP mock community was used that comprised the same 20 species plus *Porphyromonas gingivalis* str. 2561. The RBB+C protocol described by Yu and Morrison [27] was used to isolate high quality genomic DNA from all samples except the human stool and mock community. The mock community DNA was obtained from BEI Resources (catalog number HM-276D, Genomic DNA from Microbial Mock Community B, even concentration).

Vincent Young (University of Michigan) generously provided the human stool DNA from an anonymous female donor. The University of Connecticut (UConn) IRB committee determined that our research did not require IRB approval for our use of this sample as it was previously collected under an IRB approved protocol and the donor gave consent for its use in subsequent studies such as ours. The rumen and mouse samples were collected as part of IACUC approved studies being conducted at the University of Connecticut that are not a part of this current study. The UConn IACUC committee determined that this study did not require separate approval for the use of these samples as they were collected under approved protocols as part of ongoing research programs and not at the specific request of the authors. Leeches were purchased from Leeches USA, an approved supplier of medicinal leeches and termites were purchased from CT Valley Biological Supply. No specific permits or permissions were required for the acquisition of the sewage sample.

### Library preparation

We used primers previously designed to amplify the V4-V5 hyper-variable regions of the 16S rRNA gene to generate the 454 and Illumina libraries using fusion primer designs appropriate for the respective sequencing platforms (Table 1) [28]. The 16S template binding sequence was identical between both sets of fusion primers, with the 454 fusion primers following the standard format used by the Marine Biological Laboratory (MBL) and the Illumina fusion primers using the format described by Bartram et al. [17]. Libraries for all seven samples were prepared and sequenced by 454 pyrosequencing at the MBL's Josephine Bay Paul Center according to their standard protocols on a GS FLX using Titanium sequencing chemistry [28].

**Table 1:** Library construction primer sequences.

Sequencing Instrument	16S Variable Region(s)	Name	Primer Sequence 5'-3'^A^	Length
Roche 454	V4-V5	454-518F	GCCTCCCTCGCGCCATCAGXXXXX**CCAGCAGCYGCGGTA**AN	41
GS FLX		454-926R-1	GCCTTGCCAGCCCGCTCAG **CCGTCAATTCNTTTRAGT**	37
		454-926R-3	GCCTTGCCAGCCCGCTCAG **CCGTCAATTTCTTTGAGT**	37
		454-926R-4	GCCTTGCCAGCCCGCTCAG **CCGTCTATTCCTTTGANT**	37
Illumina		Iv4v5-518F	CAAGCAGAAGACGGCATACGAGATXXXXXXGTGACTGGAGTTCAGACGTGTGCTCTTCCGATCT **CCAGCAGCYGCGG**TAAN	81
MiSeq		Iv4v5-926R-1	AATGATACGGCGACCACCGAGATCTACACTCTTTCCCTACACGACGCTCTTCCGATCT *NNNN* **CCGTCAATTCNTTTRAGT**	80
		Iv4v5-926R-3	AATGATACGGCGACCACCGAGATCTACACTCTTTCCCTACACGACGCTCTTCCGATCT *NNNN* **CCGTCAATTTCTTTGAGT**	80
		Iv4v5-926R-4	AATGATACGGCGACCACCGAGATCTACACTCTTTCCCTACACGACGCTCTTCCGATCT *NNNN* **CCGTCTATTCCTTTGANT**	80
	V4	Iv4-515f	AATGATACGGCGACCACCGAGATCTACACTATGGTAATTGT**GTGCCAGCMGCCGCGGTAA**	60
		Iv4-806r	CAAGCAGAAGACGGCATACGAGATXXXXXXXXXXXXAGTCAGTCAGCC**GGACTACHVGGGTWTCTAAT**	68

A For all primers sets, the 16S template specific sequences are given in bold. For the 454 primers, the Xs in the forward represent the 5 bp run-key defined by the MBL with the underlined portion representing the 454 Lib A (forward primer) or Lib B (reverse primers) adapter sequence. Underlined portions of the Illumina primers represent the full TruSeq adapter sequence (V4-V5 primers) or a truncated version (V4). The N-bases in italics for the V4-V5 primers represent the 4 base ambiguous mix in between the TruSeq adapter sequence and the 16S template sequence. The Xs in the V4-V5 forward primer represent the sequence of one of the 6 bp TruSeq indices defined by Illumina while in the V4 forward primer they represent the 12 base Golay encoding barcode as defined by Caporaso et al.

The Illumina sequencing libraries were all prepared and sequenced at the University of Connecticut. We prepared two sets of V4-V5 Illumina libraries for the six natural community samples at two separate times. The first set of libraries was prepared following the same protocol used for the 454 pyrosequencing libraries, with the PCR product for each sample gel purified prior to pooling and sequencing. The PCR products for the second set of V4-V5 Illumina libraries and the mock community libraries were purified using a 0.6X PCR volume of AMPure XP magnetic beads following the manufacturer's instructions. Additionally, we prepared libraries for the V4 hyper-variable region according to the protocol described by Caporaso et al. [18]. The Illumina libraries were sequenced on separate runs of a MiSeq using a 2×250 bp paired end protocol.

### Sequence pre-processing

The V4-V5 454 pyrosequencing datasets were pre-processed prior to QIIME analysis in accordance with the in-house processing pipeline used by the MBL for 454 pyrosequencing analysis. Sequences had to possess the full index and forward primer sequence with no errors present in either, have zero ambiguous bases over the entire length of the read, and be longer than 300 bp after trimming of the index and forward primer sequences in order to be retained after demultiplexing with QIIME [29]. After demultiplexing, the 454 sequences were denoised using the QIIME Denoiser according to the QIIME standard protocol. The V4-V5 Illumina datasets were initially demultiplexed using MiSeq Reporter v2.0. The sequences corresponding to the forward and reverse primers were trimmed from the demultiplexed reads using cutadapt (http://code.google.com/p/cutadapt/) using similar stringency settings to those used for the 454 sequences. The trimmed read pairs were then merged into single contigs using SeqPrep (https://github.com/jstjohn/SeqPrep) followed by a length-filtering step prior to analysis with QIIME. The Illumina V4 read pairs were merged and length filtered in a similar manner as the V4-V5 reads to form single contigs prior to being demultiplexed with QIIME. Reads from all datasets were quality filtered using a *Q*20 minimum value during demultiplexing. In order to ensure an even treatment and comparison of all sequence datasets for the seven sample sources, the demultiplexed sequences for all datasets were combined and processed as a single bulk dataset for QIIME analyses.

### QIIME analysis

We used QIIME versions 1.6 and 1.7 to perform OTU clustering and alpha and beta diversity analyses [29]. Reference-based OTU clustering was done using the parallel uclust_ref method while *de novo* OTU clustering was done with standard uclust, using the default options as implemented in QIIME for both methods at the 97% similarity level. For reference OTU clustering and *de novo* OTU alignment we used the V4-V5 section of the 97% clustered Greengenes reference OTU NAST alignment [30], [31]. The 2012–10 Greengenes database release was used initially as this was the current version when analysis began. After the 2013–08 release became available, all processing was re-run with the new release, allowing us to examine the effect of the reference itself on data analysis and interpretation. Taxonomy assignments were made using the RDP Classifier after retraining against the above mentioned Greengenes reference sequences and their respective taxonomy files as recommended by Werner et al [32]. Chimera checking was performed using ChimeraSlayer with standard options as implemented in QIIME against the V4-V5 region of the Greengenes reference alignment.

A more detailed description of our creation of the V4-V5 specific Greengenes reference files and the different QIIME processing methods used is provided in the supplementary methods (File S1). The scripts (denovo.sh, Ref.sh, RDS.sh) used for QIIME analysis are also included the supplementary material (File S2).

### Data availability

The sequence data generated and used in this study were deposited in the European Nucleotide Archive SRA under project ID PRJEB4688.

## Results

**Figure 1:**
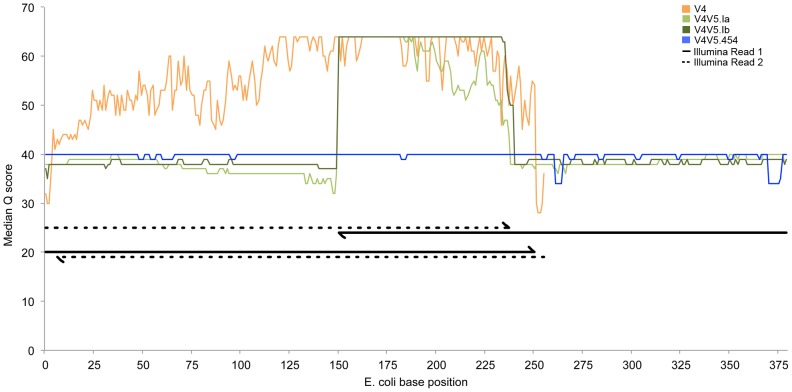
Comparison of 454 and Illumina sequence quality. Plot depicting the median per base PHRED quality scores (*Q* score) for the full length 454 and merged Illumina reads from the six natural community samples. The V4 data is shown in orange, the first V4-V5 Illumina run (V4V5.Ia) is in light green, the second run (V4V5.Ib) is in dark green, and the 454 data is in blue. The size and over-lapping regions of the V4 and V4-V5 Illumina amplicons is shown in black below the quality plots. Illumina sequencing read 1 is depicted as a solid line while read 2 is dashed, with arrow heads depicting the direction of the read in reference to the *E. coli* base position given along the X axis.

We conducted a comparison of 454 pyrosequencing and Illumina sequencing of 16S amplicons by analyzing four different sequencing libraries for six different natural microbial community samples: the V4 hyper-variable region sequenced on an Illumina MiSeq (V4.I), a V4-V5 Illumina library that was gel-purified (V4V5.Ia), a second V4-V5 Illumina library that was AMPure purified (V4V5.Ib), and a V4-V5 454 pyrosequencing library (V4V5.454). We also analyzed one V4-V5 454 library and two replicate V4 and V4-V5 Illumina libraries for a synthetic mock community. As one of the stated advantages of Illumina sequencing is a lower error rate compared to 454 pyrosequencing [15], [16], we first compared the overall quality of the sequences generated from each sequencing run. While these values represent predicted rather than absolute error rates, they are the most commonly used proxy for examining sequence quality and thus one of the primary metrics used in data pre-processing. The median PHRED quality score (*Q*-score) for each base over the length of a read had an average value of *Q*39 in the V4V5.454 datasets (Figure 1) and represented the standard to which the Illumina datasets were compared. As the error rate of Illumina sequences increases at the 3′ ends of each read, as indicated by a drop in *Q*-scores, we merged the paired Illumina reads to form a single consensus contig prior to quality and QIIME analysis. This process serves to minimize the effects of sequencing errors by forming a consensus sequence from the overlapping ends of the reads as previously demonstrated [22]. The median *Q*-score for each base of the consensus contig after read merging was similar to or greater than that of the 454 dataset (Figure 1), demonstrating that by merging the paired Illumina sequencing reads we could produce single contigs of similar length as 454 pyrosequencing but of higher average quality. Additionally, improvements to Illumina's Real Time Analysis (RTA) base-calling software that occurred during this study have resulted in significantly higher *Q*-scores for bases later in a read, which correspond to greater confidence in base-calling. This improvement can be seen in the reads from the V4V5.Ib dataset, which have higher median *Q*-scores for bases in the overlap region than the V4V5.Ia dataset, which was sequenced using an earlier version of the RTA software (Figure 1). Additional improvements from Illumina regarding MiSeq read lengths and on-instrument data analysis now suggest that merging paired reads from longer amplicons, such as those covering the V1-V3 regions are now feasible. Overall, after read merging a greater proportion of reads from the Illumina sequencing runs was retained after demultiplexing compared to the V4V5.454 data when using the same quality threshold of *Q*20 (data not shown).

### Low-levels of dataset contamination occur in Illumina sequencing

While we observed that overall read quality was higher in the Illumina datasets compared to the 454 pyrosequencing dataset, during our analyses we identified a small percentage of reads in the Illumina datasets that did not belong in the demultiplexed dataset for a given sample, a result that we did not observe in any of the 454 datasets. The source of these reads could be assigned to two separate issues that are particular to Illumina sequencing systems and especially for the MiSeq. The first source of these incorrect reads was the carry-over of samples from a previous sequencing run into the subsequent sequencing run. This occurs when samples from a previous run persist in the fluidics lines of the system and become mixed with new samples in subsequent sequencing runs [33]. If identical indices are used in consecutive sequencing runs, then the carry-over of reads from a previous library can artificially suggest the presence of low abundance OTUs that are not truly present in a subsequent sample.

The second source of incorrect reads that we identified was from other libraries that were sequenced during the same sequencing run. This was most noticeable for the V4V5.Ib datasets, which we sequenced at the same time as amplicon libraries created for non-ribosomal genes. Both the 16S V4V5.Ib and non-ribosomal libraries featured different six base TruSeq indices and we determined that ∼0.06% of reads in the 16S libraries were sequences from the non-ribosomal amplicon libraries. This was the first time that the non-ribosomal libraries were sequenced, thus the contamination could not have been due to carry-over contamination of the fluidics lines from a previous run as noted above. In addition, the 16S and non-ribosomal libraries were prepared completely independently of each other and were only pooled immediately before loading into the MiSeq, eliminating the chances of contamination during library preparation. After consultation with Illumina representatives, we assume that this result is due to sequencing and/or image analysis errors during the index sequencing phase of the MiSeq run, which occurs as a separate step in the sequencing process, and likely caused a small number of amplicons from one library to be incorrectly assigned an index corresponding to another library. While we could adequately identify the contaminating non-ribosomal sequences and remove them from our 16S datasets before proceeding with downstream analyses, this finding suggests that a similar level of index misassignment could occur between different 16S libraries when sequenced on the same run, which will artificially inflate alpha diversity measures and bias the interpretation of results when investigating low abundance OTUs.

In addition to index misassignment and/or sample carryover of similarly indexed libraries, we also identified reads from the ΦX174 (phiX) genome in all of the raw Illumina datasets. These reads originated from the unindexed phiX control library that is added to Illumina sequencing runs as an inline control library and could not have resulted from contamination during library construction. Formerly, the sequencing of 16S amplicons on the MiSeq required phiX to comprise 50–90% of the run's throughput, as was done when we sequenced the V4.I and V4V5.Ia libraries. Upgrades to the MiSeq's RTA base-calling software (since version 2.2) have reduced the amount of phiX that is recommended to be added to amplicon sequencing runs to only 2–5%, however phiX reads were still observed in the raw V4V5.Ib datasets which we sequenced with the upgraded RTA software and only 2.5% phiX. In order to prevent the presence of phiX reads in the Illumina datasets from biasing our downstream analyses, we incorporated a step in our Illumina pre-processing pipelines to identify and remove these reads prior to analysis with QIIME.

### Determining optimal OTU clustering method

**Figure 2:**
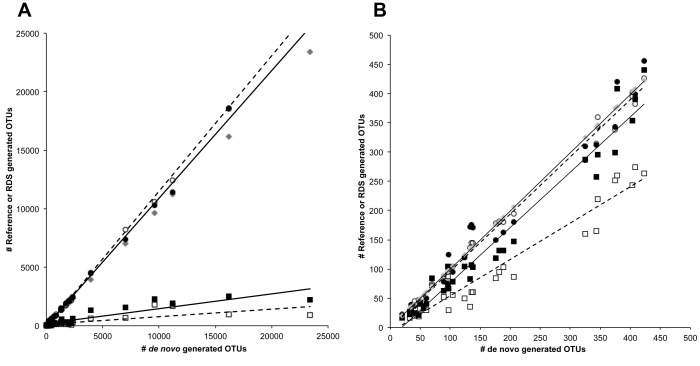
The RDS processing method replicates *de novo* OTU clustering better than reference-based clustering. The correlation between OTU clustering methods is shown by plotting the number of raw (a) and filtered (b) OTUs observed when using *de novo* OTU clustering versus reference or the RDS method. The reference-based OTU clustering results are depicted with squares while the RDS OTU clustering results are depicted with circles. Open markers indicate samples where the Greengenes 2012 reference was used while closed markers indicate samples where the Greengenes 2013 reference was used. *De novo* results are depicted as gray diamonds. Linear regression lines are shown for the reference and RDS datasets, with dash lines fitted to datasets processed using the Greengenes 2012–10 reference and solid lines fitted to datasets processed using the Greengenes 2013–08 reference.

As Illumina sequencing with the MiSeq generally produces at least 10 times more sequences than 454 pyrosequencing, recent publications discussing Illumina 16S amplicon sequencing have used and recommended reference-based OTU clustering methods to enable users to quickly process their data [18]. While reference OTU clustering has been used for the analysis of 454 data, many investigators still choose *de novo* OTU clustering methods for 454 data analysis as this method recovers OTUs not found in reference datasets. Thus we examined what effect these two clustering methods had on data analysis and the interpretation of the results. We performed *de novo* OTU clustering of the bulk dataset using standard QIIME methods for processing pyrosequencing data with uclust used for OTU clustering and chimera checking performed with ChimeraSlayer, while reference OTU clustering was performed with the parallel version of uclust_ref against the Greengenes 2012–10 reference as that was the current Greengenes release at the time. These two clustering methods yielded very different results, with the number of OTUs observed and the number of sequences assigned to an OTU being lower when performing reference-based clustering than *de novo* clustering for the same dataset (Table S1, Figure 2a).

One of the factors that contributed to the difference between the two clustering methods was that a large number of sequences failed to be assigned to a reference OTU (Table S1). On average, only 65% of the reads for a given dataset were assigned to a reference OTU, although the scale of this effect varied dramatically between the different samples. For example, over 90% of reads from each of the human stool datasets were assigned to a reference OTU, while for the termite sample only between 30% to 40% of reads from the V4-V5 datasets were assigned to a reference OTU (Table S1). As the Greengenes 2013 release occurred as we were performing our data analyses, we repeated the reference OTU clustering using this newer reference as the 2013 release includes a greater number of reference sequences than the 2012 release. When using the Greengenes 2013 release for reference OTU clustering, we observed that a greater number of sequences were assigned to a reference OTU and a greater number of OTUs per sample observed compared to using the 2012 version. Even with this improvement over the 2012 reference, we still did not observe the same number of OTUs as in the *de novo* clustered datasets (Table S1, Figure 2a). This difference in the number of OTUs based on reference or *de novo* OTU clustering carried over into the calculation of alpha diversity measures, although beta diversity analyses, particularly those using the phylogenetic tree based weighted UniFrac metric were less affected (data not shown).

### Reference plus *de novo* OTU clustering with chimera checking

In order to more closely replicate the results of *de novo* OTU clustering while retaining the processing efficiency of reference OTU clustering, we developed an analysis pipeline that first performs parallel reference OTU clustering using the 97% Greengenes OTUs as reference, followed by *de novo* OTU clustering and chimera checking with ChimeraSlayer of the sequences that failed to be assigned to a reference OTU. As discussed below, this reference plus *de novo* OTU clustering with ChimeraSlayer pipeline, which we call RDS, produced similar alpha diversity measures, taxonomic composition, and beta diversity comparison as the chimera checked *de novo* OTU clustering method. Unlike the similar open reference clustering method of uclust_ref implemented in QIIME, which can only run on a single processing core, this split implementation takes advantage of the ability to perform reference OTU clustering across multiple processing cores, reducing the total time for analysis and thus is more amenable to processing large Illumina datasets.

We compared the number of observed OTUs and the Simpson (*D*), Shannon (*H*′) and phylogenetic distance (PD) alpha diversity metrics generated by the RDS method using each of the Greengenes references to those obtained using *de novo* and reference OTU clustering. Using either Greengenes reference, the number of OTUs generated using the RDS method was more similar to the number of OTUs obtained by *de novo* clustering than for reference OTU clustering alone (Table 2, Figure 2). While the magnitude of the difference in the number of OTUs between processing methods varied for each dataset, on average the results of reference-based processing were 23.7% different from *de novo* while RDS processing was 12% different. When analyzed by ANOVA, the number of reference-clustered OTUs was significantly different from the results of *de novo* OTU clustering (p<0.01) but there was no statistical difference between the RDS method and *de novo* (p>0.05). Compared to *de novo* OTU clustering, the alpha diversity measures generated using the RDS clustering method had Pearson correlation coefficients closer to 1 and greater linear curve fits than the reference-clustered measures, indicating that the RDS method reproduces the results of *de novo* OTU clustering better than reference-based OTU clustering alone.

**Table 2:** Comparisons of alpha diversity metrics produced from different processing methods.

			*de novo*				Reference				RDS			
Sample Source	Library	Input # Seqs	# OTUs	*D*	*H'*	PD	# OTUs	*D*	*H'*	PD	# OTUs	*D*	*H'*	PD
Human stool	H.v4.I	93769	96	0.778	3.098	18.35	73	0.786	3.057	9.72	75	0.784	3.030	13.08
	H.v4v5.I.a	32506	70	0.788	3.099	11.73	84	0.793	3.224	7.58	83	0.793	3.216	10.34
	H.v4v5.I.b	153159	97	0.754	2.922	19.89	104	0.757	3.033	10.61	103	0.756	3.015	16.35
	H.v4v5.454	7882	51	0.775	2.689	11.70	42	0.772	2.624	6.98	44	0.773	2.640	8.75
Leech intestinum	L.v4.I	118954	56	0.750	2.836	12.47	33	0.611	1.903	5.56	37	0.634	2.068	9.11
	L.v4v5.I.a	44230	41	0.580	1.801	7.97	25	0.509	1.377	3.88	37	0.578	1.788	6.93
	L.v4v5.I.b6	191369	105	0.623	2.076	21.13	78	0.549	1.626	10.23	95	0.620	2.049	18.07
	L.v4v5.I.b11	171969	90	0.615	2.006	20.77	63	0.550	1.628	10.33	78	0.613	1.986	16.96
	L.v4v5.454	10229	19	0.697	2.240	6.09	17	0.676	2.085	3.04	21	0.697	2.255	5.14
HMP Mock Even	Mock.v4.I.1	213043	141	0.932	4.436	15.19	107	0.932	4.353	6.65	176	0.937	4.635	16.51
	Mock.v4.I.105	240682	143	0.936	4.574	16.43	103	0.935	4.434	6.75	171	0.941	4.777	16.19
	Mock.v4v5.I.1	2484	99	0.932	4.588	12.16	66	0.930	4.487	5.53	125	0.946	5.140	13.09
	Mock.v4v5.I.11	90126	138	0.941	4.848	14.25	83	0.943	4.672	5.96	172	0.958	5.419	15.93
	Mock.v4v5.454	7386	36	0.930	4.073	8.80	28	0.930	4.059	5.15	39	0.931	4.106	8.07
Mouse small intestine	M.v4.I	45411	61	0.743	2.620	12.59	40	0.643	1.890	7.19	50	0.653	2.010	9.60
	M.v4v5.I.a	24061	47	0.766	2.739	9.01	21	0.711	2.159	4.28	47	0.772	2.859	7.16
	M.v4v5.I.b	155976	178	0.811	3.042	39.03	132	0.764	2.469	17.87	180	0.816	3.204	35.37
	M.v4v5.454	10453	33	0.761	2.432	9.02	22	0.749	2.255	5.49	30	0.761	2.431	7.21
Rumen content	R.v4.I	93881	402	0.986	7.292	67.79	390	0.988	7.297	29.91	399	0.987	7.282	57.91
	R.v4v5.I.a	44431	372	0.984	7.176	58.58	408	0.991	7.586	25.27	420	0.991	7.650	50.96
	R.v4v5.I.b	217371	417	0.985	7.284	68.63	440	0.992	7.721	29.59	456	0.992	7.776	59.21
	R.v4v5.454	35527	323	0.985	7.161	59.15	287	0.985	7.089	26.56	310	0.986	7.209	51.04
Municipal sewage	S.v4.I	117562	375	0.955	6.389	75.57	299	0.977	6.561	30.39	343	0.952	6.247	58.70
	S.v4v5.I.a	28971	346	0.973	6.814	69.85	295	0.982	6.831	28.80	343	0.973	6.827	57.48
	S.v4v5.I.b	160654	403	0.975	6.925	80.75	354	0.984	6.982	34.40	402	0.975	6.968	66.40
	S.v4v5.454	38227	343	0.979	6.884	73.67	257	0.985	6.811	31.30	312	0.979	6.876	59.17
Termite hindgut	T.v4.I	124664	182	0.941	5.107	30.63	132	0.926	4.619	13.84	163	0.935	4.925	23.31
	T.v4v5.I.a	31220	170	0.946	5.311	27.41	119	0.937	4.968	12.95	149	0.947	5.286	22.19
	T.v4v5.I.b	164780	198	0.915	4.921	33.24	147	0.904	4.606	16.74	180	0.918	4.947	27.57
	T.v4v5.454	7146	126	0.928	4.933	25.38	104	0.920	4.679	14.06	120	0.929	4.945	21.73

Even though the RDS processing method reproduced the results of *de novo* OTU clustering better than reference-based OTU clustering according to the alpha and beta diversity measurements we examined, there were a greater number of OTUs in nearly all of the Illumina datasets than has been reported for similar samples in the literature. In particular, the Illumina datasets of the mock community had between 25 to 125 times as many OTUs as expected based on an analysis of the available reference genome sequences. One factor contributing to this increase could be the above mentioned dataset contamination, which can be partially addressed using OTU filtering strategies to remove OTUs that account for a low percentage of the total reads as recommended for Illumina datasets by Bokulich et al. [34] An analysis of different filtering methods and cutoffs showed that no single filtering value worked equally well across all samples, as cutoffs that reduced the number of OTUs in the mock community samples to reasonable numbers were overly restrictive for other samples (Table S2). Manual examination of the representative OTU sequences from the Illumina mock community datasets showed that a large proportion of them represented chimeras between two or more species from the community. Because the highly synthetic nature of the mock community is not very representative of the richness and evenness of natural samples, we chose to remove single and doubleton OTUs from the full OTU table as spurious reads, followed by filtering of OTUs representing fewer than 0.005% of all sequences as was recommended by Bokulich et al. [34] While the number of OTUs observed with reference clustering against the Greengenes 2013 reference was more similar to *de novo* after implementing the OTU filtering step, linear regression analysis showed that the RDS method still produced results more reflective of *de novo* OTU clustering (Figure 2b).

After processing the datasets using the RDS method and incorporating the OTU filtering step, the alpha diversity metrics for each of the Illumina datasets had more OTUs and a larger phylogenetic distance (PD) than the corresponding 454 dataset (Table 2). We observed a similar result when performing *de novo* OTU clustering of the datasets with the OTU filtering step. Except for one of the mock community datasets, all of the Illumina datasets for a sample had a greater number of input sequences than the corresponding 454 dataset. To prevent differences in sequencing depth from biasing our comparisons of 454 and Illumina sequencing, we normalized the number of sequences in the datasets for a sample by rarefying each dataset to the number of reads in the corresponding 454 dataset. The smallest mock community Illumina dataset was excluded from this analysis. After rarefication, the number of OTUs observed in the Illumina datasets was still greater than in the corresponding 454 dataset (Table 3) although the degree of difference was smaller for the higher diversity samples (rumen, sewage, termite) than the low diversity samples (human stool, leech, mouse).

**Table 3:** Alpha diversity measures of RDS processed samples after normalization.

Sample Source	Library	Normalized Seqs^A^	# OTUs	*D*	*H'*	PD
Human stool	H.v4.I	7737	56	0.785	2.999	9.13
	H.v4v5.I.a		72	0.793	3.232	8.95
	H.v4v5.I.b		72	0.756	3.006	9.23
	H.v4v5.454		44	0.773	2.640	8.75
Leech intestinum	L.v4.I	10213	26	0.628	2.055	6.21
	L.v4v5.I.a		28	0.575	1.767	5.40
	L.v4v5.I.b6		38	0.624	2.057	7.65
	L.v4v5.I.b11		34	0.613	1.981	6.31
	L.v4v5.454		21	0.697	2.255	5.14
HMP Mock Even	Mock.v4.I.1	7331	146	0.936	4.606	14.29
	Mock.v4.I.105		153	0.941	4.762	14.97
	Mock.v4v5.I.11		154	0.959	5.431	15.31
	Mock.v4v5.454		39	0.931	4.106	8.07
Mouse small intestine	M.v4.I	10350	34	0.650	1.993	6.37
	M.v4v5.I.a		46	0.769	2.838	7.14
	M.v4v5.I.b		55	0.815	3.196	10.30
	M.v4v5.454		30	0.761	2.431	7.21
Rumen content	R.v4.I	27672	386	0.987	7.275	55.75
	R.v4v5.I.a		420	0.991	7.650	50.96
	R.v4v5.I.b		426	0.992	7.751	53.03
	R.v4v5.454		310	0.986	7.210	51.04
Municipal sewage	S.v4.I	19354	311	0.953	6.253	52.53
	S.v4v5.I.a		343	0.973	6.827	57.48
	S.v4v5.I.b		349	0.975	6.951	58.00
	S.v4v5.454		302	0.979	6.869	58.15
Termite hindgut	T.v4.I	6850	127	0.935	4.897	18.68
	T.v4v5.I.a		136	0.949	5.338	20.10
	T.v4v5.I.b		139	0.916	4.909	19.90
	T.v4v5.454		120	0.929	4.945	21.73

A The normalized number of sequences represents the number of sequences that each dataset of a given sample were normalized to by rarefaction to allow for intra-sample comparisons of the datasets.

When we compared the number of OTUs between the V4 and V4-V5 Illumina datasets of each sample, the V4 dataset consistently had fewer OTUs than for the corresponding V4-V5 Illumina datasets. Compared to the V4-V5 amplicons, the V4 amplicons are ∼100bp shorter and cover only a single hyper-variable region. The greater number of OTUs for the Illumina V4-V5 datasets compared to the V4 after rarefaction suggests that the increased sequence information available for analysis by including the V5 hyper-variable region allowed for the discrimination of new OTUs that could not be differentiated based on the V4 region alone.

### Beta diversity analysis

**Figure 3:**
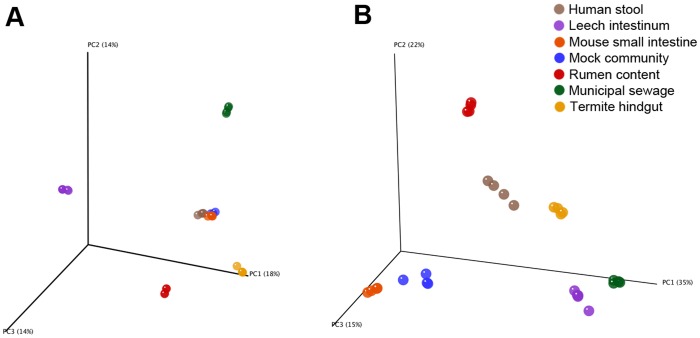
Beta diversity analysis of all datasets. Three dimensional principal coordinates analysis plots showing the relatedness of datasets using either the Bray-Curtis (A) and weighted UniFrac (B) metric. Individual datasets are represented at spheres which are colored according to their sample source as follows: human stool – brown, leech intestinum – purple, mouse small intestine – orange, mock community – blue, non-adherent rumen contents – red, mixed liquor – green, termite hindgut – gold.

Beta diversity analysis of all datasets showed that each sample source represented a distinct microbiome irrespective of the processing method used. Each of the individual datasets clustered together on the basis of their original sample source as determined by principal coordinates analysis of the Bray-Curtis and UniFrac distances between each dataset (Figure 3). This clustering was independent of the hyper-variable regions chosen for sequencing, V4 or V4-V5, or the sequencing platform used, GS FLX or MiSeq, indicating that these factors had no obvious effect on the interpretation of beta diversity analyses when comparing the diverse group of samples we used in this study. While the RDS method did not produce beta diversity results identical to those generated when using *de novo* OTU clustering, the overall interpretation of results was similar between the two methods. The primary difference that we observed was that when using the Bray-Curtis metric the human, mouse, and mock community samples were shown to be more similar to each other when the data was processed using the RDS method compared to using *de novo* OTU clustering. This result was similar to what we observed when performing reference OTU clustering only, and suggests that these three samples shared a greater percentage of OTUs as a result of the reference OTU clustering step of the RDS method than we observed with *de novo* clustering (Figure S1).

Because of the large overall dissimilarities between the seven samples as determined by principal coordinate analysis, we also performed beta diversity analyses of the datasets for each sample independently. In each case, the V4 Illumina dataset was consistently more different from the corresponding V4-V5 datasets than the V4-V5 datasets were from each other, indicating that choice of hyper-variable region had a greater effect on beta diversity than the choice of sequencing technology (Figure S2).

### Effects of hyper-variable region and OTU clustering method on observed taxonomic diversity

While alpha and beta diversity measures provide important insights into the structure and relationship of microbial communities, a key aspect of generating hypotheses about the functional and physiological aspects of a microbial community is knowing its taxonomic composition. We determined the effect of the hyper-variable region chosen for sequencing and of the OTU clustering method used for analysis on the taxonomic composition of a sample by comparing the taxonomy summaries for each dataset when processed using *de novo*, reference-based, and the RDS OTU clustering methods. These comparisons revealed that for some samples there was a large effect on the observed taxonomic composition of the choice of hyper-variable regions sequenced or OTU clustering method used.

**Figure 4:**
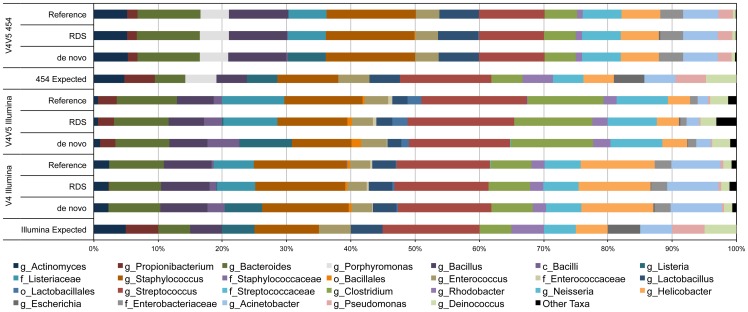
Effect of processing method on the taxonomic composition of the mock community datasets. Plot comparing the taxonomic composition of the mock community sample for the three different library types sequenced when processed three different ways. The replicate V4 and V4-V5 Illumina datasets were combined into one representative dataset for each library type. All taxonomic assignments were made using the RDP Classifier after retraining with the 2013-08 Greengenes reference. Taxonomic ranks are noted by letters preceding the taxon name as follows: genus – g, family – f, order – o.

We tested the three processing pipelines using control DNA from a synthetic mock community created as part of the Human Microbiome Project (HMP) to determine if processing method alone introduced a source of bias [7]. The mock community DNA used for the Illumina libraries comprised 20 cultured bacterial species, while the DNA used for the 454 library also included *Porphyromona gingivalis*. None of the resulting datasets showed a taxonomic composition that was identical to the known composition of the mock community, however each of the three processing methods (*de novo*, reference, RDS) yielded a similar taxonomic composition for each of the three types of libraries (V4.I, V4V5.I, and V4V5.454, Figure 4).

The abundance of some taxa was affected dramatically by the type of library that was created and the processing method. In the V4 Illumina libraries, the genus *Propionibacterium* was almost completely absent while in the V4-V5 libraries it represented ∼1.5% of the 454 dataset and 2.4–2.9% in the Illumina datasets. This result was likely due to primer specificity of the V4 primers compared to the V4-V5 primers, as there is a single base pair difference between the V4 forward primer and the annealing site based on the *P. acnes* reference genome. Across all three library types, we consistently observed that the genus *Listeria* was only identified when using *de novo* OTU clustering, whereas the family *Listeriaceae* was instead observed when using the reference or RDS processing methods. Similarly, the genus *Escherichia* was only marginally identified in any of the datasets regardless of processing, with the family *Enterobacteriaceae* instead being the predominant taxonomic assignment for these OTUs. It is interesting to note that this result only occurred when the Greengenes 2013 release was used for taxonomy assignment, as OTUs were correctly classified as *Escherichia* when we used the 2012 version of the reference.

**Figure 5:**
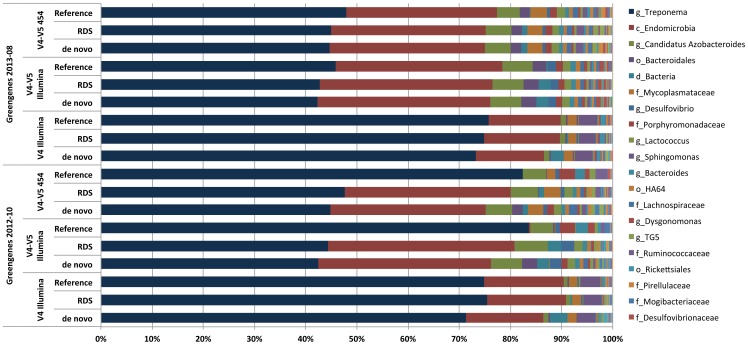
Effects of processing method and Greengenes database version on the taxonomic composition of the termite datasets. Plot comparing the taxonomic composition of the termite hindgut sample for the three different library types sequenced when processed using three different methods. The replicate V4 and V4-V5 Illumina datasets were combined into one representative dataset for each library type. Taxonomic assignments were made using the RDP Classifier after retraining with either the 2012–10 or 2013–08 Greengenes references. Taxonomic ranks are noted by letters preceding the taxon name as follows: genus – g, family – f, order – o, class – c, phylum – p, domain – d.

While the taxonomic composition of the mock community datasets showed little to no specific bias associated with the choice of hyper-variable regions sequenced or data processing method used, we did observe some distinct differences in the six natural microbial community samples that we analyzed. During our initial analyses using the Greengenes 2012 reference for OTU clustering and taxonomic assignment we observed that for certain samples the use of reference clustering alone often missed entire taxa. The most dramatic example of this was with the V4-V5 libraries for the termite sample, for which the class *Endomicrobia* was almost completely absent from the reference clustered datasets but comprised nearly 30% of the community when using *de novo* or the RDS processing methods (Figure 5). While this issue was largely resolved with the Greengenes 2013 release, the taxonomic composition of the RDS processed datasets were more similar to the *de novo* OTU clustered datasets than the reference clustered datasets were. The termite sample was also where differences between the V4 and V4-V5 libraries were the most apparent. While the V4-V5 Illumina and 454 libraries were not statistically different from each other, the V4 library was significantly different from both V4-V5 libraries (data not shown). In the V4-V5 libraries the genus *Treponema* comprised ∼45% of the community but nearly 75% in the V4 library regardless of processing method (Figure 5). While we could not determine the exact cause of this discrepancy from the data, it is possible that primer amplification bias contributed to this result.

In the human stool sample datasets, the abundance of the two most abundant genera, *Bacteroides* and *Escherichia*, differed greatly between each of the three library types (Figure S2). While the differences between the V4 and V4-V5 libraries are likely due to the choice of different primers, the genus *Bacteroides* was more abundant in the V4-V5 Illumina libraries compared to the 454 (∼65% vs. 55%), while *Escherichia* was much less abundant (∼10% vs. ∼28%). This difference in abundance between the two V4-V5 library types was observed even after normalization of the datasets by rarefaction, and thus does not directly represent a sampling depth bias. As noted above for the mock community datasets, the genus *Escherichia* was only identified in datasets processed using the Greengenes 2012 reference for taxonomy assignment, with a single exception of the V4-V5 Illumina datasets processed using the RDS method. In this case, the genus *Escherichia* was observed when using the 2013 reference but not at the same level as when using the 2012 (Figure S2). We observed similar differences in taxonomic composition of the sample corresponding to library type for the other five natural community samples that we analyzed, although these differences were minor for the rumen and sewage datasets which had the highest overall taxonomic diversity of the seven samples we analyzed.

## Discussion

### Illumina sequencing can faithfully supplant 454 pyrosequencing

The primary goal of this study was to examine how well Illumina sequencing could serve as a direct replacement for 454 pyrosequencing while using existing 16S sequencing primers and analysis workflows. To determine this we analyzed six natural microbial communities and a mock community by using both 454 pyrosequencing and Illumina sequencing of the V4-V5 hyper-variable region of the 16S rRNA gene. We additionally performed Illumina sequencing of the V4 region using the protocol developed by Caporaso et al. [18], which has been adopted as the standard protocol for Illumina 16S sequencing by researchers participating in the Earth Microbiome Project. Because the individual reads generated with the MiSeq are shorter than the single reads generated by the GS FLX, and previous studies [22] and our own analysis found that error rates increased towards the 3′ end of the reads, we utilized read merging of the paired Illumina reads to create single consensus Illumina reads with similar length to those generated by 454 sequencing. This pre-processing step for the Illumina datasets yielded merged reads that had a higher average quality than for the reads generated by 454 pyrosequencing (Figure 1), along with a greater number of reads per sample (Table 2).

When analyzing all of the datasets from the samples *en masse* we observed small differences in alpha diversity measures between the pyrosequencing and Illumina datasets for the high diversity samples while larger differences were observed for low diversity samples. Conversely, PCoA plots of beta diversity analyses showed that there was little to no apparent effect of the sequencing method used (454 or Illumina) or variable regions chosen (V4 or V4-V5), as each dataset from a given sample clustered together (Figure 3). Analysis of the individual datasets for each sample, however, did reveal that the V4 dataset was consistently more different from the V4-V5 datasets than the V4-V5 datasets were from each other (Figure S2). Part of this difference stems from the use of primers that anneal to different regions of the 16S rRNA gene for library creation, which likely have different amplification biases and template specificity [35], [36]. This bias was apparent in examining the taxonomic composition of the mock community datasets which all had slightly different abundances for each taxon across the three library types we examined and the genus *Propionibacterium* nearly absent from the V4 libraries. While we did observe differences between the V4-V5 454 and V4-V5 Illumina datasets, these differences did not significantly affect the overall interpretation of beta-diversity analyses, although their effect on taxonomic composition varied according to sample. On the basis of our overall findings, we can conclude that researchers who wish to switch to Illumina sequencing from 454 pyrosequencing should be able to modify their existing primers by simply replacing the 454 adaptor sequences with Illumina TruSeq adaptor sequences. An additional option for researchers who do not need or wish to adapt a pre-established 454 workflow is to use one of the published V4 sequencing formats developed for Illumina sequencing by Caporaso et al. or Kozich et al.[18], [22] While choosing a different hyper-variable region for analysis did affect the results in a sample-dependent manner, our analyses show that overall the V4 amplicons produced similar alpha and beta diversity measures as the V4-V5 amplicons.

One of the major differences between the Roche 454 GS FLX and Illumina MiSeq instruments is that the MiSeq is currently capable of generating well over 10 times as many sequence reads as the GS FLX in a single sequencing run. Combined with much lower operating costs, Illumina sequencing on the MiSeq provides researchers with the opportunity to sequence individual samples to a greater sampling depth than is feasible with the GS FLX and/or to include more samples in a single sequencing run through increased multiplexing of barcoded libraries. As sequencing depth increases however, a greater number of erroneous sequences can be incorporated into the resulting dataset, which will artificially bias estimates of alpha diversity through the generation of spurious OTUs. These erroneous sequences often arise due to chimera formation and PCR errors during library preparation, or are the result of sequencing errors that were not identified and removed during data processing. Protocols have been developed for 454 pyrosequencing to minimize the presence and effects of illegitimate sequences/OTUs on diversity analyses, and we incorporated these protocols as appropriate into our library preparation and data processing and analysis methods [37]–[39].

To minimize the effects of sequencing errors we first merged the paired Illumina reads to form a single consensus sequence prior to OTU clustering. This step results in a higher confidence that the base calls for the merged region are correct and thus reduces sequencing associated errors in the Illumina datasets (Figure 1). We also incorporated chimera checking with ChimeraSlayer as part of our RDS analysis pipeline. However, as demonstrated with the Illumina-sequenced mock community samples, not all chimeric OTUs were correctly identified and removed. One reason for this is that the chimera checking process typically depends on comparing differences in the sequence similarity of the two ends of a query sequence to two or more reference sequences derived either from a reference database such as Greengenes or chosen from within the dataset itself. This method poses a problem in detection as chimeras present in short sequences from closely related organisms are more difficult to identify than in longer sequences. Additionally, chimeric sequences originating from three or more parent sequences, such as those observed in the Illumina mock community datasets, may not be identified as chimeric but as novel sequences instead.

### Reference OTU clustering can bias observed diversity

As the volume of sequence data generated by Illumina instruments is orders of magnitude greater than for the GS FLX, processing and analysis pipelines that were designed to handle pyrosequencing datasets have had to be modified to process Illumina data more efficiently. One such modification has been a shift from using *de novo* generation of OTUs for large sequencing datasets to the use of reference OTUs such as those from the Greengenes [30], [31] or Silva [40] reference databases. The principal advantage of reference OTU clustering is that it is significantly faster than *de novo* OTU generation as it can be run in parallel across multiple processing cores, and the availability of reference datasets with pre-constructed phylogenetic trees and taxonomies allows for a simplified and more efficient analysis pipeline. However, with reference-based OTU clustering alone the observed microbial diversity of a sample can only be as diverse as the reference set itself, which can artificially limit the observed diversity for highly diverse or exotic environments whose microbial populations have few representative sequences in reference databases.

In this study we found that performing reference-based OTU clustering using either the Greengenes 2012 or 2013 references resulted in a reduction in the number of observed OTUs compared to *de novo* OTU clustering (Tables 2 and S1, Figure 2). The use of reference-based OTU clustering alone also had large effects on the observed taxonomy some samples, with certain taxa completely missing or misidentified when reference clustering was used compared to *de novo* (Figure 5). While the curators of the Greengenes database have made great efforts to expand their reference datasets to include more sequences from highly diverse and complex microbial communities, our results suggest that additional improvements are needed to provide better coverage for many non-human associated microbial environments. This is of particular importance as a greater number of researchers take advantage of the low cost of Illumina sequencing to characterize the microbial communities in many new and diverse environments that may not be well represented in current reference databases.

As we demonstrated, one option that researchers have is to perform reference OTU clustering and then to analyze the reduced number of sequences that did not match the reference data set using *de novo* OTU clustering, which we described above as the reference plus *de novo* with ChimeraSlayer, or RDS method. Our results demonstrated that the RDS method produces alpha (Table 2) and beta (Figure 2) diversity metrics and taxonomy summaries (Figure 3) that are more similar to *de novo* OTU clustering than reference-based clustering alone. While the current open reference picking implementation of uclust_ref allows for the creation of *de novo* OTUs from reads not assigned to a reference sequence, this process is limited to running on a single processing core. Our implementation of two separate steps for reference and *de novo* OTU clustering in the RDS method allows for reference clustering to be performed across multiple processing cores. This hybrid analysis method allows researchers to efficiently analyze large sequencing datasets generated with Illumina sequencing platforms while retaining the ability to identify novel OTUs that are not currently present in reference datasets.

### Limitations of reference databases for taxonomy assignment

While not always feasible, *a priori* knowledge of the general composition of a microbial community can provide important checks for validating the results of high-throughput 16S sequencing surveys. Our inclusion of the mock community developed by the Human Microbiome Project partially served as such a control to identify potential issues with our library construction, sequencing and data analysis workflows. When using the Greengenes 2012 reference that was available when we began this study, we found that the taxonomic composition of the mock datasets differed greatly from expected, with many OTUs not being classified to the genus level but to higher taxonomic ranks instead. The release of the 2013–08 Greengenes reference database resolved many of these assignment issues, however the genus *Escherichia* was still not correctly identified when performing taxonomic assignment of OTUs with the Greengenes 2013 reference and the genus *Listeria* was only identified in the *de novo* processed datasets.

During our initial analysis of the leech intestinum samples using the Greengenes 2012 reference no OTUs were assigned to the genus *Aeromonas* for any of the datasets regardless of processing method, a finding inconsistent with previous culture and non-culture based studies we conducted of the leech intestinum [41], [42]. We subsequently determined that this was due to a lack of any sequences in the Greengenes reference being annotated to the genus *Aeromonas*, with the lowest taxonomic rank being the family *Aeromonadaceae*. After communicating this and other findings to the Greengenes curators, an updated reference taxonomy was released (Greengenes 2013–08) that included additional genus and species level annotations compared to the previous release. However, even after performing taxonomic classifications with this updated reference only one OTU, representing less than 0.2% of all sequences in the V4-V5 datasets, was classified as *Aeromonas* when using the RDS method while all other OTUs were classified as *Aeromonadaceae* (data not shown). It is important to note that while this classification is not technically incorrect, it is less informative about the composition of the community and can potentially lead to inaccurate conclusions in situations where *a priori* knowledge of a microbial community is unknown.

This example also highlights the need for wider community efforts to ensure the highest possible accuracy of large reference datasets such as Greengenes. As the current version of the Greengenes database comprises over 1 million individual sequences, it is extremely challenging for the manual and automated curation steps to successfully identify and remove all potential chimeric sequences and ensure accurate taxonomic assignments for all sequences in the database. While this had a noticeable effect on the taxonomic composition of the leech intestinum, it appeared to have little to no effect on the composition of the human stool, rumen and sewage samples. Our findings suggest that researchers who rely on a reference dataset, such as for OTU clustering or taxonomy assignment as we do with the RDS processing method, should take caution in the interpretation of their results.

### Low levels of cross-contamination in Illumina datasets

While our results show that overall Illumina and 454 pyrosequencing produced similar alpha and beta diversity results, we did observe cases of dataset contamination that appear to be specific to Illumina of 16S amplicons. For libraries sequenced at the same time, we also observed instances of index misassignment that resulted in a small percentage of reads from one library being incorrectly assigned an index sequence corresponding to a different library. This was most apparent when we sequenced the V4-V5.Ib libraries at the same time as non-ribosomal amplicon libraries. The source of index misassignment likely arises from image analysis errors during the index sequencing phase of the run, which may be addressed by future upgrades to the MiSeq software, hardware, or reagent kits. Reducing the target cluster density for amplicon libraries below Illumina's recommended values may reduce the occurrence of this error, while also improving read quality as previously discussed by Kozich et al. [22]. The use of dual indexing formats where indices are present at both ends of the amplicon being sequenced would likely decrease the occurrence of index misassignment, as errors would need to occur in both indices in order for a read to be assigned to the incorrect sample. We also observed a low percentage of reads from the phiX control library in all of the raw Illumina datasets we used in this study. While updates to the MiSeq's RTA base-calling software have reduced the potential for phiX reads to be incorrectly assigned a valid index sequence they did not eliminate it. We removed phiX reads from the datasets prior to QIIME analysis by applying the pre-processing methods detailed above. An additional concern with Illumina sequencing that we did not directly quantify with our datasets is low-levels of carryover contamination that occurs between consecutive MiSeq runs. This issue was acknowledged in a technical bulletin from Illumina which quantified the level of carryover contamination as typically being less than 0.1% of reads for a run being carried over into and contaminating a subsequent run [33].

The combination of index misassignment occurring at a rate of ∼0.06% and carryover contamination between MiSeq runs of less than 0.1% can provide a baseline value that serves as a threshold to help distinguish which results stem from true biological signal and which may be due to noise. In order to mitigate index misassignment and sample carryover contamination for experiments that require high levels of sensitivity, we have begun to include one or more indexed control samples to more accurately quantify this occurrence. These control libraries can be created from a synthetic template, pure culture, or mock community and serve as inline controls for determining the level of index misassignment that occurs between different samples within a run and carryover contamination across separate sequencing runs. It is also recommended to alternate the indices used between runs to further reduce potential carryover contamination in high-sensitivity experiments. While researchers who are primarily concerned with identifying broad changes in microbial composition will typically not be affected by index misassignment and carry-over contamination, implementing the above listed suggestions will improve the quality and accuracy of amplicon sequencing datasets produced on Illumina instruments. Researchers focused on examining the “rare biosphere” or the role of low abundant organisms in a community may need to implement additional precautions.

Our analysis shows that primers designed for Roche 454 instruments can be readily modified for use on Illumina instruments and produce consistent results. When we utilized the same template primers, the Illumina-produced datasets were more similar to the 454-produced datasets than when different template primers were used. The consistency between platforms was further improved by using the RDS processing pipeline, maximizing the quality of the sequences by merging of the paired Illumina reads, and minimizing artifacts due to the use of reference datasets and the inclusion of chimera checking. To account for and reduce the low levels of index misassignment and carryover contamination that we observed, we recommend the use of control libraries and alternating indices between consecutive sequencing runs when using the MiSeq. Overall our results show that Illumina sequencing of 16S rRNA genes is a cost effective approach that can readily supplant 454 pyrosequencing as the new standard analysis method for microbial populations.

## Supporting Information

Figure S1 **Effects of processing method on PCoA analysis using the Bray-Curtis metric.**
(PDF)

Figure S2 **Effect of processing method and Greengenes reference on taxonomic composition of the human stool sample.**
(PDF)

Table S1 **Comparison of the number of OTUs and retained reads using different processing methods.**
(PDF)

Table S2 **Comparison of OTU filtering cut-off values.**
(PDF)

File S1 **Supplementary QIIME processing methods.**
(DOCX)

File S2 **Archive containing the processing scripts used in for this study.**
(ZIP)
