# Supplementary material for: Analysis, Optimization and Verification of Illumina-Generated 16S rRNA Gene Amplicon Surveys
Source: PLoS One. 2014 Apr 10;9(4):e94249. doi: 10.1371/journal.pone.0094249 (PMC3983156; doi:10.1371/journal.pone.0094249)
Supplement: Figure S1 — Effects of processing method on PCoA analysis using the Bray-Curtis metric. (PDF) [file pone.0094249.s001.pdf]

Figure S1: Effects of processing method on PCoA analysis using the Bray-Curtis metric

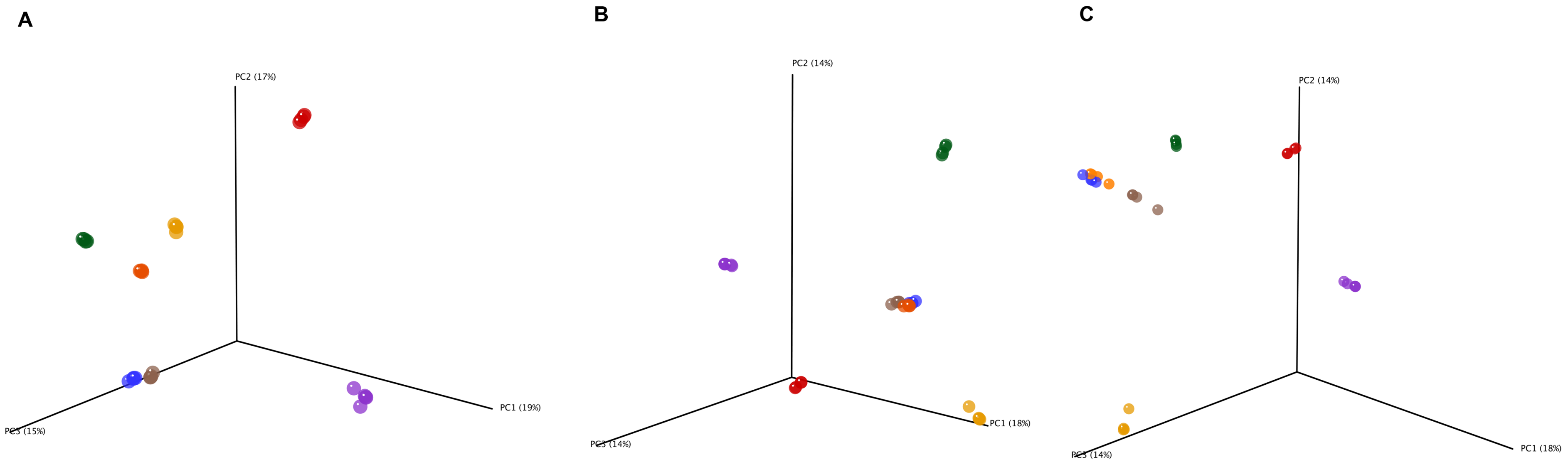

Three dimensional principal coordinates analysis plots showing the relatedness of datasets using the Bray-Curtis metric after processing by *de novo* OTU clustering (A), RDS (B), or reference-based OTU clustering (C). Individual datasets are represented at spheres which are colored according to their sample source as follows: human stool – brown, leech intestinum – purple, mouse small intestine – orange, mock community – blue, non-adherent rumen contents – red, mixed liquor – green, termite hindgut – gold.
