# Supplementary material for: Analysis, Optimization and Verification of Illumina-Generated 16S rRNA Gene Amplicon Surveys
Source: PLoS One. 2014 Apr 10;9(4):e94249. doi: 10.1371/journal.pone.0094249 (PMC3983156; doi:10.1371/journal.pone.0094249)
Supplement: Figure S2 — Effect of processing method and Greengenes reference on taxonomic composition of the human stool sample. (PDF) [file pone.0094249.s002.pdf]

Figure S2: Effect of processing method and Greengenes reference on taxonomic composition of the human stool sample

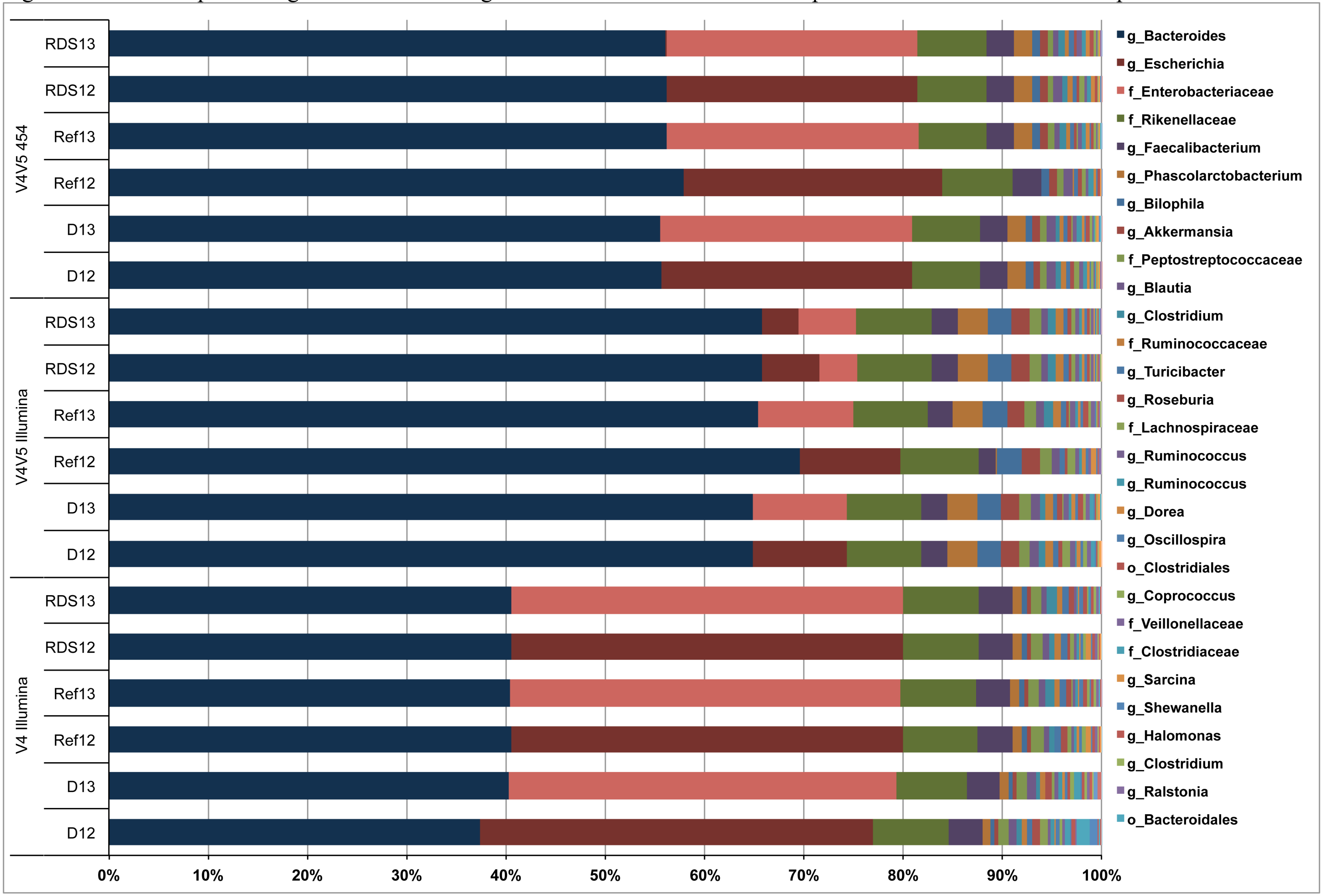

Plot comparing the taxonomic composition of the termite hindgut sample for the three different library types sequenced when processed using three different methods: *de novo* (D), reference-based (Ref), and RDS. The replicate V4 and V4-V5 Illumina datasets were

combined into one representative dataset for each library type. Taxonomic assignments were made using the RDP Classifier after retraining with either the 2012-10 or 2013-08 Greengenes references as denoted by the numbers 12 or 13 following the processing ID. Taxonomic ranks are noted by letters preceding the taxon name as follows: genus – g, family – f, order – o, class – c, phylum – p, domain – d.
