# Supplementary material for: Analysis, Optimization and Verification of Illumina-Generated 16S rRNA Gene Amplicon Surveys
Source: PLoS One. 2014 Apr 10;9(4):e94249. doi: 10.1371/journal.pone.0094249 (PMC3983156; doi:10.1371/journal.pone.0094249)
Supplement: Table S1 — Comparison of the number of OTUs and retained reads using different processing methods. (PDF) [file pone.0094249.s003.pdf]

Table S1: Comparison of the number of OTUs and retained reads using different processing methods

Sample Source	Sample ID	Initial # Reads	*de novo* % Reads Retained	*de novo* # OTUs	Reference GG-2012 % Reads Retained	Reference GG-2012 # OTUs	Reference GG-2013 % Reads Retained	Reference GG-2013 # OTUs	RDS GG-2012 % Reads Retained	RDS GG-2012 # OTUs	RDS GG-2013 % Reads Retained	RDS GG-2013 # OTUs
Human stool	H.v4.I	93769	99.65%	457	99.17%	303	99.24%	367	99.85%	564	99.92%	594
	H.v4v5.I.a	32506	99.79%	327	92.24%	138	98.63%	256	99.77%	429	99.88%	462
	H.v4v5.I.b	153159	99.98%	269	93.99%	179	99.84%	363	99.96%	406	99.99%	456
	H.v4v5.454	7882	99.96%	71	96.28%	40	99.47%	62	99.81%	70	99.92%	74
Leech intestinum	L.v4.I	118954	99.23%	301	95.19%	73	95.58%	90	99.41%	291	99.20%	284
	L.v4v5.I.a	44230	99.36%	135	63.27%	27	91.31%	42	99.24%	139	99.35%	148
	L.v4v5.I.b6	191369	99.28%	316	63.81%	80	90.07%	134	99.26%	386	99.29%	372
	L.v4v5.I.b11	171969	99.61%	275	64.61%	81	91.43%	127	99.58%	345	99.62%	329
	L.v4v5.454	10229	99.92%	23	74.89%	18	96.38%	21	99.90%	27	99.92%	26
Mouse small intestine	M.v4.I	45411	98.67%	268	96.49%	145	96.96%	166	99.08%	299	99.21%	288
	M.v4v5.I.a	24061	97.64%	188	52.39%	37	86.60%	59	97.93%	206	98.80%	206
	M.v4v5.I.b	155976	98.18%	696	58.26%	149	85.94%	286	97.82%	819	98.92%	785
	M.v4v5.454	10453	99.80%	46	74.71%	23	96.96%	32	99.24%	47	99.59%	47
HMP Mock Even	Mock.v4.I.1	213043	99.29%	1325	92.54%	152	92.97%	294	99.07%	1349	99.36%	1495
	Mock.v4.I.105	240682	98.98%	1708	90.13%	163	90.79%	312	98.73%	1766	99.10%	1933
	Mock.v4v5.I.1	2484	95.33%	449	65.26%	36	69.57%	96	95.57%	440	95.41%	472
	Mock.v4v5.I.11	90126	96.71%	2250	67.20%	64	71.27%	237	95.93%	2185	96.03%	2329
	Mock.v4v5.454	7386	100.00%	39	98.82%	28	98.86%	32	99.80%	50	99.96%	52
Rumen content	R.v4.I	93881	92.61%	11215	64.78%	1700	70.71%	1930	94.77%	12457	94.33%	11413
	R.v4v5.I.a	44431	93.03%	7051	42.29%	702	67.28%	1542	94.43%	8241	93.40%	7414
	R.v4v5.I.b	217371	91.35%	23398	37.86%	908	62.42%	2236	92.73%	27493	91.40%	25385
	R.v4v5.454	35527	98.11%	1339	55.68%	278	82.88%	584	98.52%	1474	97.81%	1413
Municipal sewage	S.v4.I	117562	93.03%	9628	57.94%	1797	62.21%	2271	95.01%	10605	94.83%	10271
	S.v4v5.I.a	28971	93.71%	3925	35.11%	644	59.29%	1352	95.01%	4526	94.95%	4496
	S.v4v5.I.b	160654	91.65%	16167	32.45%	961	54.70%	2534	93.05%	18573	92.78%	18629
	S.v4v5.454	38227	94.72%	2315	41.39%	292	65.11%	637	95.63%	2463	95.68%	2460
Termite hindgut	T.v4.I	124664	94.89%	2040	79.78%	235	85.59%	307	96.31%	2181	97.48%	2095
	T.v4v5.I.a	31220	98.72%	853	36.37%	117	85.73%	219	98.46%	960	99.17%	854
	T.v4v5.I.b	164780	98.67%	1949	31.09%	138	87.32%	323	98.30%	2111	99.02%	2087
	T.v4v5.454	7146	98.46%	193	40.39%	63	90.61%	139	98.10%	193	98.46%	196
