# Supplementary material for: Analysis, Optimization and Verification of Illumina-Generated 16S rRNA Gene Amplicon Surveys
Source: PLoS One. 2014 Apr 10;9(4):e94249. doi: 10.1371/journal.pone.0094249 (PMC3983156; doi:10.1371/journal.pone.0094249)
Supplement: Table S2 — Comparison of OTU filtering cut-off values. (PDF) [file pone.0094249.s004.pdf]

Table S2: Comparison of OTU filtering cut-off values

Sample Source	Library	Raw	0.001%	0.005%	0.01%	0.05%	0.1%	0.5%
Human stool	H.v4.I	594	155	74	53	24	20	8
	H.v4v5.I.a	462	151	82	50	25	19	8
	H.v4v5.I.b	456	187	102	70	37	27	12
	H.v4v5.454	74	61	44	31	17	12	6
Leech intestinum	L.v4.I	284	66	36	31	18	12	5
	L.v4v5.I.a	148	72	37	30	17	9	6
	L.v4v5.I.b6	372	149	95	78	52	34	16
	L.v4v5.I.b11	329	126	77	66	41	27	14
	L.v4v5.454	26	25	21	20	16	12	8
HMP Mock Even	Mock.v4.I.1	1495	427	172	106	38	29	14
	Mock.v4.I.105	1933	430	167	103	37	28	14
	Mock.v4v5.I.1	472	245	124	76	30	23	11
	Mock.v4v5.I.11	2329	476	169	100	35	27	14
	Mock.v4v5.454	52	49	39	32	23	19	11
Mouse small intestine	M.v4.I	288	84	50	39	20	17	13
	M.v4v5.I.a	206	79	47	35	16	9	7
	M.v4v5.I.b	785	250	180	156	75	48	19
	M.v4v5.454	47	39	30	22	12	7	5
Rumen content	R.v4.I	11413	1258	392	214	43	21	2
	R.v4v5.I.a	7414	1308	410	216	38	17	3
	R.v4v5.I.b	25385	1403	446	247	59	31	12
	R.v4v5.454	1413	622	302	181	38	18	1
Municipal sewage	S.v4.I	10271	1006	336	197	47	28	10
	S.v4v5.I.a	4496	1006	338	194	42	20	6
	S.v4v5.I.b	18629	1153	396	236	69	40	17
	S.v4v5.454	2460	708	310	191	53	30	10
Termite hindgut	T.v4.I	2095	333	161	120	51	34	14
	T.v4v5.I.a	854	302	147	110	43	27	9
	T.v4v5.I.b	2087	358	178	137	57	37	11
	T.v4v5.454	196	166	119	89	36	22	7
