# Supplementary material for: Analysis, Optimization and Verification of Illumina-Generated 16S rRNA Gene Amplicon Surveys
Source: PLoS One. 2014 Apr 10;9(4):e94249. doi: 10.1371/journal.pone.0094249 (PMC3983156; doi:10.1371/journal.pone.0094249)
Supplement: File S1 — Supplementary QIIME processing methods. (DOCX) [file pone.0094249.s005.docx]

Supplementary methods:

Creation of the V4-V5 specific Greengenes reference files:

Werner et al. [32] previously demonstrated that the use of reference sequences corresponding to the sequenced hyper-variable region(s) improved the accuracy of reference-based methods such as sequence alignment and taxonomic classification. To create a region specific reference for this study we took the NAST aligned 97% clustered reference OTUs of the Greengenes database and located the position of the forward and reverse primers within the alignment. Using a custom perl script, we cut the alignment based on these positions to excise the V4-V5 region from the full alignment, including 15 base-pairs up and down stream to ensure the sequenced amplicons would lie within the reference. Common gap columns were removed from the extracted alignment to reduce the size of the aligned V4-V5 reference sequence file, which improves computational efficiency for reference-based alignment and chimera checking. Alignment characters were stripped from the aligned V4-V5 reference file to produce the unaligned set of reference sequences that were used for reference-based OTU clustering and training of the RDP Classifier. This was done initially for the 2012-10 release and subsequently for the 2013-08 release when it was made available.

Description of QIIME analysis pipelines:

Multiple QIIME processing methods were analyzed in order to determine an analysis pipeline that optimized both accuracy and processing efficiency of large Illumina datasets. Final versions of the shell scripts that include the OTU filtering step that we used for running the *de novo* (denovo.sh), reference-based (Ref.sh), and RDS (RDS.sh) pipelines are provided as part of the supplementary material as a single compressed archive (.zip) file (File S2).

A brief description of each pipeline follows.

*De novo* processing pipeline: For our initial processing with *de novo* OTU clustering, the combined demultiplexed sequence dataset was clustered using uclust at the 97% similarity level. Representative sequences for each OTU were chosen and then aligned with NAST against the aligned V4-V5 reference file. The aligned representative sequences were then chimera checked using ChimeraSlayer against the aligned V4-V5 Greengenes reference described above. Any OTUs for which the representative sequence failed alignment or were marked as potential chimeras were excluded from further analysis. Taxonomic assignment of OTUs was made based on the representative sequence using the RDP Classifier, after retraining it with the unaligned V4-V5 Greengenes reference and the appropriate taxonomy file as recommended by Werner et al. [32] A phylogenetic tree was constructed using FastTree as implemented in QIIME from the aligned OTU representative sequences.

Reference-based processing pipeline: Reference-based OTU clustering was carried out using the parallel implementation of uclust_ref as implemented in QIIME and the unaligned V4-V5 Greengenes reference. Representative sequences for each OTU were chosen from the aligned Greengenes reference sequence file and alignment characters were stripped to create an unaligned set or representative sequences. No chimera checking was performed on the reference-based OTUs under the assumption that the reference sequences were non-chimeric. Taxonomic assignments were made similarly to the method used for the *de novo* OTU processing pipeline.

Reference plus *de novo* OTU clustering with chimera checking (RDS): As a large number of sequences failed to be assigned to a reference OTU, we analyzed a third processing pipeline that combined parallel reference-based OTU clustering with *de novo* OTU clustering of the reads that were not assigned to a reference OTU. The first step of this method conducts parallel OTU clustering with uclust_ref against the V4-V5 Greengenes reference. Representative sequences for the reference-clustered OTU were selected from the aligned V4-V5 Greengenes reference, while all sequences that failed to be assigned to a reference OTU were then collected into a new file for *de novo* OTU picking. Representative sequences for the *de novo* OTUs were selected from the collected sequences unassigned to a reference OTU and aligned using NAST against the V4-V5 Greengenes reference and chimera checked using ChimeraSlayer as described above for As for the *de novo* pipeline, any *de novo* OTUs for which the representative sequence failed alignment were excluded from further analysis. The results of the reference-based and *de novo* OTU clustering steps were merged to create a single, unified set of OTUs and aligned and un-aligned representative sequences. Taxonomy assignment and phylogenetic tree construction were conducted as described for the *de novo* pipeline.

For each processing method, beta diversity analyses were conducted after first normalizing the filtered OTU table to the smallest dataset in the study, excluding the V4V5.I.1 mock community dataset. Per sample analyses of each microbial community were conducted after creating sample specific OTU tables from the original filtered OTU table. The sample specific OTU tables were then normalized to the smallest dataset of each sample, again excluding the V4V5.I.1 mock community dataset.
